# Parasitism of *Aedes albopictus* by *Ascogregarina taiwanensis* lowers its competitive ability against *Aedes triseriatus*

**DOI:** 10.1186/s13071-021-04581-0

**Published:** 2021-01-25

**Authors:** Emma Stump, Lauren M. Childs, Melody Walker

**Affiliations:** 1 Systems Biology, Virginia Tech, Hahn Hall South Suite 2108, 24061 Blacksburg, VA USA; 2 Department of Mathematics, Virginia Tech, 460 McBryde Hall, 225 Stanger Street, 24061 Blacksburg, VA USA

**Keywords:** Mosquito population dynamics, Competition, Aedes albopictus, Aedes triseriatus, Parasitism, Ascogregarina taiwanensis, Ascogregarina barretti

## Abstract

**Background:**

Mosquitoes are vectors for diseases such as dengue, malaria and La Crosse virus that significantly impact the human population. When multiple mosquito species are present, the competition between species may alter population dynamics as well as disease spread. Two mosquito species, *Aedes albopictus* and *Aedes triseriatus*, both inhabit areas where La Crosse virus is found. Infection of *Aedes albopictus* by the parasite *Ascogregarina taiwanensis* and *Aedes triseriatus* by the parasite *Ascogregarina barretti* can decrease a mosquito’s fitness, respectively. In particular, the decrease in fitness of *Aedes albopictus* occurs through the impact of *Ascogregarina taiwanensis* on female fecundity, larval development rate, and larval mortality and may impact its initial competitive advantage over *Aedes triseriatus* during invasion.

**Methods:**

We examine the effects of parasitism of gregarine parasites on *Aedes albopictus* and *triseriatus* population dynamics and competition with a focus on when *Aedes albopictus* is new to an area. We build a compartmental model including competition between *Aedes albopictus* and *triseriatus* while under parasitism of the gregarine parasites. Using parameters based on the literature, we simulate the dynamics and analyze the equilibrium population proportion of the two species. We consider the presence of both parasites and potential dilution effects.

**Results:**

We show that increased levels of parasitism in *Aedes albopictus* will decrease the initial competitive advantage of the species over *Aedes triseriatus* and increase the survivorship of *Aedes triseriatus*. We find *Aedes albopictus* is better able to invade when there is more extreme parasitism of *Aedes triseriatus*. Furthermore, although the transient dynamics differ, dilution of the parasite density through uptake by both species does not alter the equilibrium population sizes of either species.

**Conclusions:**

Mosquito population dynamics are affected by many factors, such as abiotic factors (e.g. temperature and humidity) and competition between mosquito species. This is especially true when multiple mosquito species are vying to live in the same area. Knowledge of how population dynamics are affected by gregarine parasites among competing species can inform future mosquito control efforts and help prevent the spread of vector-borne disease. 
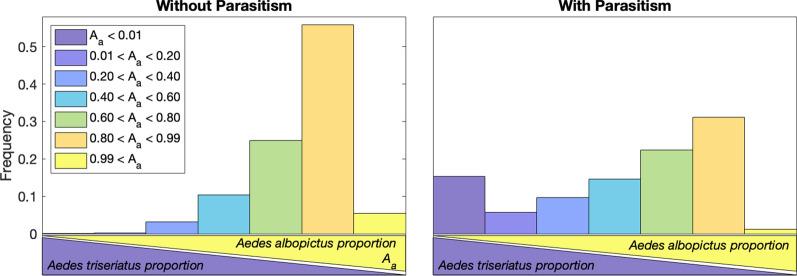

## Background

La Crosse encephalitis virus (LACV) can cause brain swelling and lead to severe neuroinvasive disease in children under 15 [1], which can cause lifelong health effects [2]. While it only leads to a few reported cases each year, it is likely under-reported. We focus on two species known to transmit LACV, *Aedes albopictus* and *Aedes triseriatus*, and the competition between them [3, 4]. Female mosquitoes of these species may become disease vectors when they feed on infected individuals and transmit the virus to other animals or humans via a second bloodmeal [5].

*Aedes (Ae.) albopictus*, also known as the Asian tiger mosquito, is native to subtropical regions of southeast Asia and is a common vector of arboviruses such as Dengue fever virus, West Nile virus, and La Crosse encephalitis virus [6]. The first appearance of *Ae. albopictus* in the USA was in Houston, Texas, in 1985 [7, 8]. Since its introduction, *Ae. albopictus* has spread throughout the continental US and now occupies much of the southern and eastern US, stretching from Texas to New Jersey [9]. *Ae. albopictus* is known for its ability to adapt to a range of climates and to be a strong competitor to other species that share larval habitats [10]. In 2001, La Crosse virus was isolated in *Ae. albopictus* [11]. Fourteen years later, it was estimated that 3.01 of every 1000 *Ae. albopictus* were infected with the La Crosse virus [12].

*Ae. triseriatus*, known as the eastern tree hole mosquito, is native to the eastern US. *Ae triseriatus* is the primary vector for the La Crosse virus [3, 4], a pathogen endemic to southwest Virginia [2, 3]. *Ae. albopictus* will bite both small mammals and humans, making it more likely to infect human’s than *Ae. triseriatus*, whose preference is for small mammals [4].

Several laboratory studies have shown that *Ae. albopictus* is the superior competitor compared to *Ae. triseriatus* [13–19]. In Bevins [14], they showed a 10% drop in survival for *Ae. triseriatus* when a quarter of the mosquitoes present were *Ae. albopictus* and a 20% drop in *Ae. triseriatus* survival when half of the mosquitoes were *Ae. albopictus*. In Ho et al. [13], the authors showed that while the development time of *Ae. albopictus* was not significantly altered by competition, the development time of *Ae. triseriatus* was increased when in a shared habitat with other *Aedes* species. Moreover, *Ae. albopictus* larvae inhibit egg hatching of other species such as *Ae. triseriatus*, while there is no significant inhibition on their own species [15]. However, in a more recent meta-analysis, competitive equivalence of *Ae. albopictus* and *Ae. triseriatus* was suggested [20]. In a study with two different environments, they found that *Ae. albopictus* did much better in tires, but did worse than *Ae. triseriatus* in treeholes [16].

*Ae. albopictus* and *Ae. triseriatus* are parasitized by *Ascogregarina (As.) taiwanensis* and *Ascogregarina (As.) barretti*, respectively. *Ascogregarina* are intestinal protozoan parasites that inhabit the gut of the mosquito throughout its life cycle [7, 21]. The effects and prevalence of these parasites are heterogeneous. In the wild, typically 67 to 95% of a given population of *Ae. albopictus* are infected with *As. taiwanensis* [22]. Infection of *Ae. albopictus* by *As. taiwanensis* lengthens larval development time for both male and female mosquitoes, reduces adult female fecundity, increases larval mortality, and reduces egg laying and hatching rates [7, 23]. However, Aliabadi and Juliano saw that mortality of *Ae. albopictus* was not significantly affected by *As. taiwanensis* [7]. For *Ae. triseriatus*, one study found 80% of their collected sites harbored *As. barretti*. Treeholes showed more infected sites than tires [24]. In contrast, another study found that only 5 and 23.6% of *Ae. triseriatus* are infected [25]. Development time and mortality have been seen to increase in *Ae. triseriatus* when infected by *As. barretti* [21, 25]. However, Beier and Harris [24] showed no significant effect of *As. barretti* on *Ae. triseriatus* mortality. While *As. barretti* impacts the fitness of *Ae. triseriatus*, its effects are mostly dependent on resource availability [21]. In low resource conditions they find that the *Ae. triseriatus* development time is increased significantly, but with sufficient resources there is not a significant difference. Another study showed a significant difference in survival of *Ae. triseriatus* infected with *As. barretti* compared to those uninfected [25].

In this work, we build a mathematical model of *Aedes* population dynamics accounting for parasitism of *Ae. albopictus* by *As. taiwanensis* and *Ae. triseriatus* by *As. barretti*. Our model formulation encodes competition between the two species of *Aedes* using the Lokta-Volterra model, a basis for many inter-specific competition models [26–31] including mosquito populations [16, 32–34]. In Kuno [35], a two-species Lokta-Volterra competition model is introduced, which includes reproductive interference. Other competition models also show the importance of reproductive interference between *Ae. aegypti* and *Ae. albopictus* [34, 36]. However, in contrast to the reports on reproductive interference seen with *Ae. albopictus* and *Ae. aegypti*, there appears to be a lack of studies evaluating reproductive interference between *Ae. albopictus* and *Ae. triseriatus*. Livdahl and Willey [16] compared *Ae. albopictus* and *Ae. triseriatus* using a Lokta-Volterra model to explain how possible competition affects them in either tree holes or tires. This model was then extended to include La Crosse spread [32] and showed a lack of importance of *Ae. albopictus* in the recent resurgence of LACV.

**Fig. 1:**
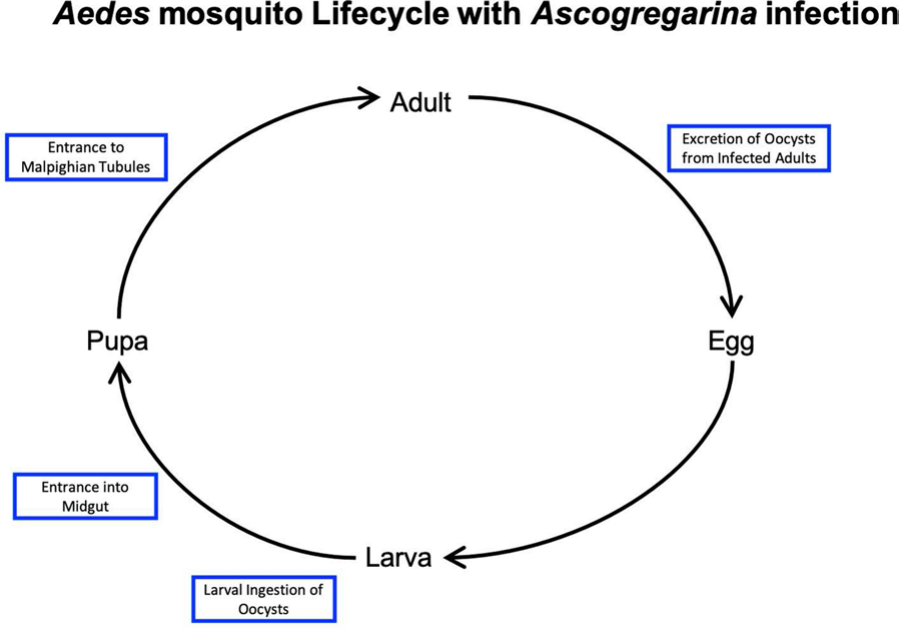
Life cycle of the mosquito and parasite. Interaction between life cycles of *Aedes* mosquito, shown as the black lines, and *Ascogregarina* parasite, shown as the blue boxes. As the mosquito progresses from stage to stage in its life cycle, its gregarine parasite progresses through in a mirrored fashion. For details on the parasite life cycle, see Chen [37]

The life cycle of gregarine parasites mimics that of their host: each stage of its development is analogous to a stage within mosquito development. See the inset in Fig. 1. Transmission of gregarine parasites is horizontal between *Aedes* mosquitoes individuals and does not occur from parent to offspring [22]. Initial infection occurs when the mosquito larvae ingest oocysts. After ingestion, the gregarine parasite travels through the midgut, epithelial tissues, and excretory system of the mosquito, transitioning into different life stages as the mosquito matures to adulthood. From there, the parasite reproduces and offspring are excreted by the adult mosquitoes into breeding containers [37, 38].

Since new *Ae. albopictus* habitats are unlikely to be contaminated with *As. taiwanensis*, new populations of *Ae. albopictus* can initially escape parasitism. This lack of parasite infection in newly established populations gives the mosquito a potential competitive advantage over *Ae. triseriatus* [7, 22]. Knowledge of how parasite infection affects the survivorship of *Ae. albopictus* will provide insight into the competition between the two mosquito species and subsequently help to deepen understanding of the spread of mosquito-borne infectious disease. In this study, we examine whether the parasitism of *Ae. albopictus* by *As. taiwanensis* decreases the mosquito’s competitive ability enough to alter the dynamics between the two *Aedes* mosquitoes. We build a compartmental model of the interactions of the two mosquito species and the effect of the parasites *As. taiwanensis* and *As. barretti*. We parameterize the model from published literature, simulate dynamics, investigate a wide parameter space, and examine the effects of parasitism.

## Methods

We constructed a four-compartment model of mosquito population dynamics including larval and adult stages of both *Ae. albopictus* and *Ae. triseriatus* (Eq. 1). We simplify the life cycle of the *Aedes* mosquito to just two stages because most, if not all, of the effects of competition and parasitism are felt at the larval and adult stages. Furthermore, including all mosquito life stages would unnecessarily complicate the model. We based our model of competition on the Lotka-Volterra model of species interaction [39–42]. We use the Lokta-Volterra model to incorporate inter-specific competition between the two species and include effects of parasitism. We assume a fixed level of parasitism in *Ae. triseriatus*, impacting development time and mortality. We consider two conditions: either a high or low effect of *As. barretti*. To model the effect of different *As. taiwanensis* parasite levels as *Ae. triseriatus* becomes established, we vary the level parasitism. We incorporate the effect of parasitism on *Ae. albopictus* in three ways: (i) decreased fecundity, (ii) increased development time, and (iii) increased larval mortality. This provides a potential decrease in the competitive advantage of *Ae. albopictus* over the population of *Ae. triseriatus* as *Ae. albopictus* becomes established in a new area.

The model includes four compartments: larval *Ae. albopictus* ($L_a$), adult *Ae. albopictus* ($A_a$), larval *Ae. triseriatus* ($L_t$), and adult *Ae. triseriatus* ($A_t$). Larval mosquitoes $L_i$ are born at rate $\beta _i$, die at rate $\mu _{L_i}$, and develop at rate $\delta _i$ where $i\in \{a,t\}$. Adults emerge from larva at rate $\delta _i$ and die at rate $\mu _{A_i}$ where $i\in \{a,t\}$ for *Ae. albopictus* and *Ae. triseriatus*, respectively. Similar formulations for *Ae. albopictus* population dynamics are found in [43]. The parameters $\rho _a$ and $\rho _t$ represent the proportion of adults that are female. *K* is the carrying capacity of the larval population for the two species. The Lotka-Volterra competition coefficients, $\alpha _a$ and $\alpha _t$, quantify the intrinsic effect of one species on the other. Parasitism for *Ae. albopictus* is included through the parameters representing effects on female fecundity ($\gamma _{b_a}$), larval development rate ($\gamma _{d_a}$), and larval mortality ($\gamma _{m_a}$). The parasite parameters for *Ae. triseriatus* are larval development rate ($\gamma _{d_t}$) and larval mortality ($\gamma _{m_t}$). This forms our system of ordinary differential equations:

1 $$\begin{aligned} \frac{dL_a}{dt}= & {} \frac{\beta _a}{\gamma _{b_a}}\rho _a A_a \left( 1- \frac{L_a + \alpha _tL_t}{K}\right) - \frac{1}{\gamma _{d_a}\delta _a} L_a - \gamma _{m_a} \mu _{L_a} L_a, \nonumber \\ \frac{dA_a}{dt}= & {} \frac{1}{\gamma _{d_a}\delta _a} L_a - \mu _{A_a} A_a,\nonumber \\ \frac{dL_t}{dt}= & {} \rho _t \beta _t A_t \left( 1- \frac{\alpha _aL_a + L_t}{K}\right) - \frac{1}{\gamma _{d_t}\delta _t} L_t - \gamma _{m_t}\mu _{L_t} L_t,\nonumber \\ \frac{dA_t}{dt}= & {} \frac{1}{\gamma _{d_t}\delta _t} L_t - \mu _{A_t} A_t. \end{aligned}$$

From a literature search, we determined biologically relevant ranges for parameter values. A description of the parameters is provided in Table 1. As it was often difficult to directly find relevant values in the literature, we transformed values found to meet our parameter descriptions. The birthrate was calculated from data for the gross reproductive rate (GRR) and length of gonotrophic cycle (GC) by taking the minimum GRR and dividing by the maximum length of the GC. The maximum value was found by dividing the maximum GRR by the minimum GC length [44]. With this, we calculated a range of 2.5–56 eggs laid per adult female per day. A similar calculation was performed to find a birthrate for *Ae. triseriatus*, where we calculated a range of 3–26 eggs laid per adult female per day [45, 46]. Development time can vary based on a variety of factors and has been found to be as short as 9 days and as long as 40 days for *Ae. albopictus* [13, 47, 48]. *Ae. triseriatus* has been shown to develop more slowly than *Ae. albopictus* [13, 18]. In the model, these values are incorporated directly as development time of larvae, ($\delta _a$) and ($\delta _t$). Larval ($\mu _{L_a}$) and adult ($\mu _{A_a}$) mortality for *Ae. albopictus* were calculated to be 0.067 and 0.05, respectively, from survival rates and development rates [13, 44, 47, 48], such that $\mu = 1-($ survival rate$)^{(1/\delta )}$. For *Ae. triseriatus*, larval ($\mu _{L_t}$) and adult ($\mu _{A_t}$) mortalities were found to be 0.009 and 0.1, respectively [49, 50]. The carrying capacity for the larval population of both species (*K*) was set at 60 as was used for a study performed in 200 ml of water [7]. It is important to note that this number is relative and could be scaled to fit different size larval containers. In this study we focus on the proportion of each species such that raw population sizes are not influential. There is evidence of potential sex imbalance among mosquitoes especially under low food resources. Thus, for *Ae. triseriatus*, we set the proportion of females, $\rho _t$, to be between 0.2 and 0.6 [17]. In the same study, *Ae. albopictus* showed less variation, $0.4-0.55$, in the proportion females, so we set $\rho _a$ accordingly.

**Table 1:** Parameter values and Latin hypercube sampling ranges

*Aedes albopictus*					
Symbol	Description	Value	LHS range	Units	Reference
$\beta _a$	Birth Rate	32.6	2.5–56	Eggs/day	[44]
$\delta _a$	Development Time	10	9–45	1/days	[13, 47, 48]
$\mu _{L_a}$	Larval mortality	0.067	0.005–0.4	1/day	[13, 44]
$\mu _{A_a}$	Adult mortality	0.045	0.01–0.065	1/day	[44, 47, 48]
$\alpha _a$	Competition parameter	0.83, 0.42	0.4–1	Unitless	[16]
$\rho _a$	Adult female proportion	0.5	0.2–0.6	Unitless	[17]
*Aedes triseriatus*					
$\beta _t$	Birth rate	11	3–26	Eggs/day	[45, 46, 50]
$\delta _t$	Development time	22	13–55	1/days	[12, 13, 18],
$\mu _{L_t}$	Larval mortality	0.009	0.002–0.011	1/day	[13, 17, 48]
$\mu _{A_t}$	Adult mortality	0.1	0.03–0.1	1/day	[49]
$\alpha _t$	Competition parameter	0.25, .73	0–0.75	Unitless	[16]
$\rho _a$	Adult female proportion	0.5	0.4–0.55	Unitless	[17]
Other parameters					
$\gamma _{b_a}$	Effect on fecundity	1.0	1–2	Unitless	Varied
$\gamma _{d_a}$	Effect on development rate	1.0	1–2	Unitless	Varied
$\gamma _{m_a}$	Effect on larval mortality	1.0	1–16	Unitless	Varied
$\gamma _{d_t}$	Effect on development rate (*Ae. Triseriatus*)	1.2, 2	Fixed	Unitless	Varied
$\gamma _{m_t}$	Effect on larval mortality (*Ae. Triseriatus*)	1.5, 4	Fixed	Unitless	Varied
*K*	Carrying capacity	60	Fixed	Number of larvae	[16]

In Livdahl and Willey [16], the competition parameters from the Lokta-Volterra model were fit using nutrient fluids. The competition parameter of *Ae. albopictus* on *Ae. triseriatus* ($\alpha _a$) and the competition parameter of *Ae. triseriatus* on *Ae. albopictus* ($\alpha _t$) were found to be 0.42 and 0.73 in treehole fluid and 0.83 and 0.25 in tire fluid, respectively. Many factors dictate which species will be the better competitor; however, as we considered that *Ae. albopictus* is generally the greater competitor, we chose a range of [0, 0.75] for the effect on *Ae. albopictus* from *Ae. triseriatus* and a range of [0.4, 1] for the effect on *Ae. triseriatus* from *Ae. albopictus*.

The effects of parasitism for *Ae. albopictus* on female fecundity ($\gamma _{b_a}$), larval development rate ($\gamma _{d_a}$), and larval mortality ($\gamma _{m_a}$) were shown to vary with resource availability and environmental context [7, 23, 25, 51]. In Comiskey et al. [23], they found that in low resource conditions the mortality of infected *Ae. albopictus* larvae was seven times greater than in uninfected larvae. They also found that fecundity and fertility were reduced by > 20% and development time was increased by 44%. In Aliabadi and Juliano [7], they showed that the development time of infected *Ae. albopictus* increases with greater interspecific competition with *Ae. triseriatus*, whereas without *Ae. triseriatus*, the median development time of infected *Ae. albopictus* was not significantly different. They also found that survival rates were significantly different for *Ae. triseriatus* at lower densities with *Ae. albopictus*. They did not observe a significant change in survival for *Ae. albopictus* from parasitism, but a greater effect from intra- and inter-specific competition. *Ae. triseriatus* was found in one study to have as much as a 2.8–3.5 greater death rate when infected [25]. In a relatively recent study by Soghigian and Livdahl [51], they showed that in the absence of parasite infection, survival of *Ae. albopictus* is about 98%, but with the greatest amount of infection observed, the survival decreases to approximately 71%. This constitutes a 15 times greater mortality rate. In another study by Walker et al. [21], they found an increase in development time for *Ae. triseriatus* in the laboratory with low resources, but not in the field experiment.

We initially set all *Ae. albopictus* parasite parameter values to 1, which represents the case with no parasitism. We then consider ranges from 1 to 2 for a parasite’s effect on fecundity and development time and 1–16 for mortality. These values were chosen to cover the ranges of changes found in [23, 51]. We will extend the ranges of the parasite parameters for development time and fecundity in further analyses. We are assuming that *Ae. triseriatus* is already infected, so we initially fix these parameter values as $\gamma _{d_t} = 1.2$ and $\gamma _{m_t} = 1.5$ to be a relatively small effect. We also consider a higher effect to parasitism with $\gamma _{d_t} = 2$ and $\gamma _{m_t} = 4$ as a comparison. If $\gamma _{d_a} = 5$, this would mean that the development time was five times greater when *Ae. albopictus* is infected with its parasite. If any of the parasite parameters are set to 1 this indicates no effect of parasitism.

We simulated the mosquito population dynamics from our ODE model in Matlab. Fixed parameter values, shown in Table 1, were used in these simulations. The initial conditions used for all simulations were $L_a = 30, A_a = 0, L_t = 30$, and $A_t =0$. We use these initial conditions for consistency with Aliabadi and Juliano [7], but as we run the simulation until equilibrium other initial values will give the same results. Simulations were run for 2000 days, long enough for the population of *Ae. albopictus* and *Ae. triseriatus* larvae and adults to reach equilibrium. To begin, we considered two competition scenarios. The first was the environment within a tire ($\alpha _a=0.83$, $\alpha _t=0.25$), which indicates that *Ae. albopictus* has a greater effect on *Ae. triseriatus*. In the second scenario, the environment in a treehole ($\alpha _a = 0.42$, $\alpha _t = 0.73$), such that *Ae. triseriatus,* has a greater effect on *Ae. albopictus*. These parameters were from fitted data in Livdahl and Willey [16].

We performed a parameter sweep by sampling ranges of the parameters using Latin Hypercube Sampling (LHS) in Matlab [52]. We conducted this with a sample size of 100,000, and sampled 12 of the parameters: $\beta _a$, $\beta _t$, $\delta _a$, $\delta _t$, $\mu _{L_t}$, $\mu _{A_t}$, $\mu _{L_a}$, $\mu _{A_a}$, $\alpha _t$, $\alpha _a$, $\rho _t$, and $\rho _a$. Values for parasitism on *Ae. albopictus* ($\gamma _{b_a}$, $\gamma _{d_a}$, $\gamma _{m_a}$) were fixed at 1, and *K* was fixed at 60. The LHS was completed twice, first with the parasite parameters for *Ae. triseriatus* set at $\gamma _{d_t} =1.2$ and $\gamma _{m_t} =1.5$ and then again with them set at $\gamma _{d_t} =2$ and $\gamma _{m_t} =4$. With the results generated by the LHS, we generated a histogram reflecting the type of outcomes for each of the samples. We categorized the results into seven categories, described in Table 2. These categories were based on the proportion of the population occupied by *Ae. albopictus* as follows: in category (1), a proportion of < 0.01; in category (2), a proportion of 0.01 to 0.2; in category (3), a proportion between 0.2 and 0.4; in category (4), a proportion between 0.4 and 0.6; in category (5), a proportion between 0.6 and 0.8; in category (6), a proportion between 0.8 and 0.99; in category (7) a proportion > 0.99. We than did a sensitivity analysis on the parameters to account for uncertainty and variation in parameters by the partial rank correlation coefficient [53].

After sampling the space of the 12 parameters in the model in the absence of parasitism in *Ae. albopictus*, we focused on investigating the effects of the parasite. We repeated the LHS including the parasite parameters for *Ae. albopictus*. We also performed sensitivity analysis on the 100,000 samples, which included varied parasite effects. The mortality and fecundity parasite parameters were varied from 1 to 4, and the development time parasite parameter varied from 1 to 16, where a value of 1 meant the parasite has no effect and a value of 3 meant a three-fold increase of the associated parameter value. This means that mortality or development time is increased or fecundity is decreased compared to the value without parasitism.

To examine the varying effects of parasitism, all parameters were fixed as listed in Table 1 except we varied the three *Ae. albopictus* parasite parameters with *Ae. triseriatus* fixed at either a high or low parasite effect. We compared effects on female fecundity, larval development rate, and larval mortality to see how these different combinations led to different outcomes in terms of the proportion *Ae. albopictus* in the final population. We extended the range of the parasite parameters so that all were varied from 1 to 10.

**Table 2:** Categorized outcomes $^{\sharp }$Criteria is proportion of *Ae. albopictus* adults

Category	Description	Criteria$^{\sharp }$ ($A_a$)
1	*Ae. triseriatus* completely dominates, Ae. albopictus wiped out	$A_a < 0.01$
2	*Ae. triseriatus* dominates, few *Ae. albopictus* remain	$0.01< A_a < 0.15$
3	*Ae. triseriatus* more present, *Ae. albopictus* still persists	$0.20< A_a < 0.40$
4	Both species coexist in even proportions	$0.40< A_a < 0.60$
5	*Ae. albopictus* more present, *Ae. triseriatus* persists	$0.60< A_a < 0.80$
6	*Ae. albopictus* dominates, few *Ae. triseriatus* remain	$0.80< A_a < 0.99$
7	*Ae. albopictus* completely dominates, *Ae. triseriatus* wiped out	$A_a > 0.99$

### Dilution effects

A recent study by Westby et al. [54] showed that with *Ae. japonicus* and *Ae. triseriatus* there was a dilution effect on the amount of parasitism. *Ae. japonicus* decreased the number of *As. barretti* by consuming the parasite and not propagating it [54]. We consider this idea with the two-species model, by allowing the parasitism parameters to have decreased effect in the presence of greater proportions of the competing species, a so-called ‘dilution’ effect. We modify the model by making all parasite parameters a function of the proportion of the total population. Specifically, each parasite parameter will linearly decrease from the maximum value $\gamma _{i_{max}}$ to 1. This is captured by:

2 $$\begin{aligned} \gamma _i(p)= & {} (\gamma _{i_{{max}}}- 1)p + 1 \end{aligned}$$

where *p* is the proportion of a species. We choose a linear function for simplicity. Notice that if $p = 1$, $\gamma _ i = \gamma _{i_{max}}$, which indicates that if a particular species is 100% of the population, then their parasite parameters will be at its maximum. If $p =0$, $\gamma _ i = 1$, this means that as the species goes to 0% of the population, the parasite effect of the parameter will linearly decrease to no parasite effect. In Westby et al. [54], they see a large decrease in the amount of parasitism ($\approx$ 82% reduction), so we allow parasitism parameters to approach 1 as the proportion of larvae goes to zero. Recall that, when the parasite parameters equal 1, this represents no effect of parasitism. Thus, our revised system of equations becomes:

$$\begin{aligned} \frac{dL_a}{dt}= & {} \frac{\beta _a\rho _a A_a}{\gamma _{b_a}(P_a)} \left( 1- \frac{L_a + \alpha _tL_t}{K}\right) - \frac{1}{\gamma _{d_a}(P_a)\delta _a} L_a - \gamma _{m_a}(P_a) \mu _{L_a} L_a, \\ \frac{dA_a}{dt}= & {} \frac{1}{\gamma _{d_a}(P_a)\delta _a} L_a - \mu _{A_a} A_a, \\ \frac{dL_t}{dt}= & {} \rho _t \beta _t A_t \left( 1- \frac{\alpha _aL_a + L_t}{K}\right) - \frac{1}{\gamma _{d_t}(P_t)\delta _t} L_t - \gamma _{m_t}(P_t)\mu _{L_t} L_t, \\ \frac{dA_t}{dt}= & {} \frac{1}{\gamma _{d_t}(P_t)\delta _t} L_t - \mu _{A_t} A_t. \end{aligned}$$

where $$P_a = \frac{L_a}{L_a+L_t}$$ and $$P_t = \frac{L_t}{L_a+L_t}$$.

We then consider four cases by choosing different maximum parameters for parasitism, $\gamma _{i_{{max}}}$. These combination are: *Ae. albopictus* and *Ae. triseriatus* both have minor effects of parasitism, both have more severe effects, and only one has a severe effect and the other minor effect. See Table 3 for specific choices for maximum parasite parameters.

## Results

**Fig. 2:**
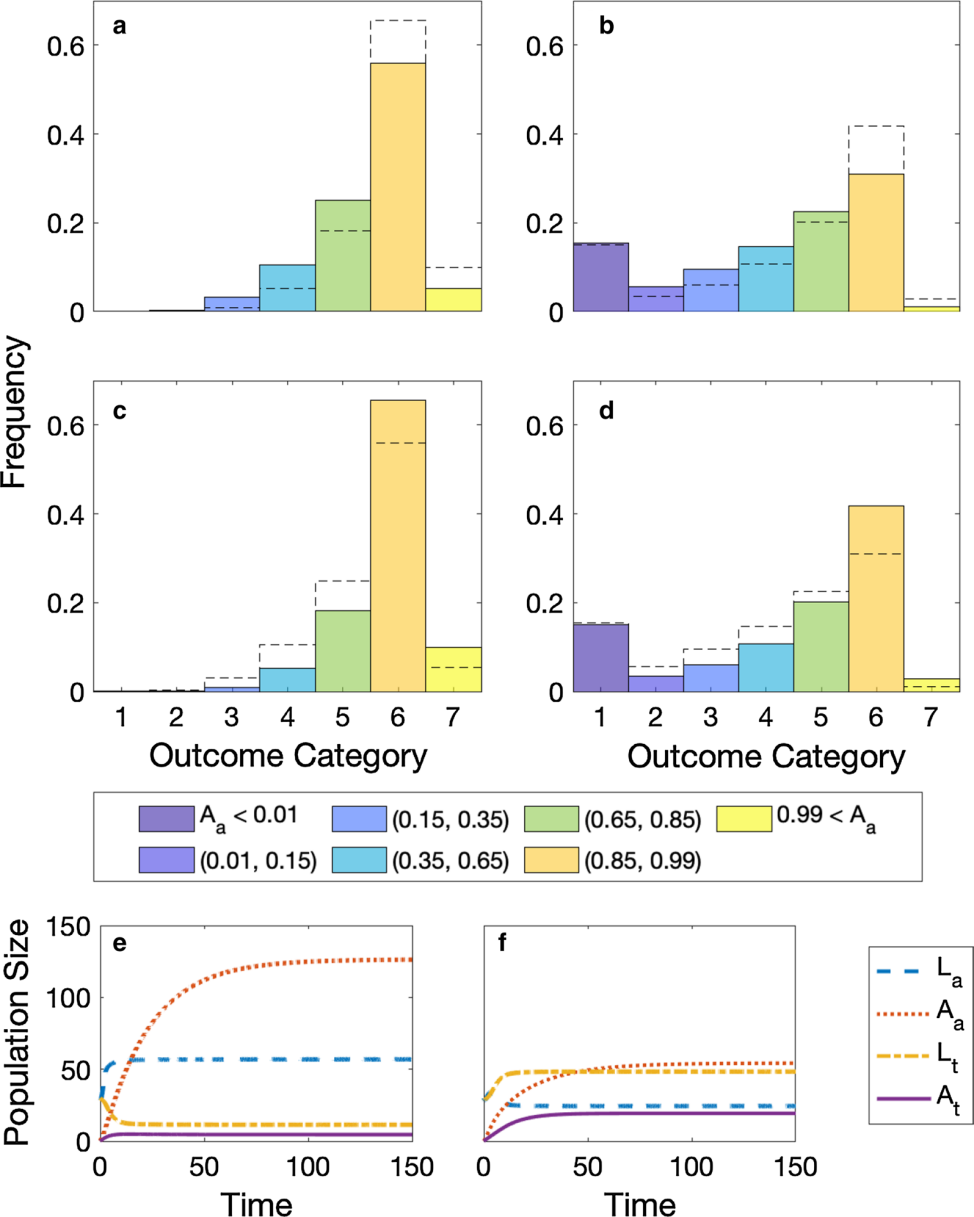
Competition outcomes in Latin Hypercube Sampling. The outcomes of competition with the 100,000 samples from the LHS. The categories shown in dark blue have *Ae. albopictus* ($A_a$) wiped out and the bright yellow is when *Ae. albopictus* completely dominates *Ae. triseriatus*. The left (**a**, ** c**) represents without effects of parasitism on *Ae. albopictus* and the right (**b**, ** d**) includes parasitism effects. The top row (**a**, **b**) has a low parasite effect, and the middle row (**c**, **d**) has a high parasite effect on *Ae. triseriatus*. The black dashed lines in (**a**–**d**) are for reference to compare low versus high density, (**a**–**c**) and (**b**–**d**). Categories are defined in Table 2. The dynamics plots (**e**,** f**) are the expected temporal results without any parasitism in *Ae. albopictus* and the low effect of parasitism in *Ae. triseriatus*. This represents when *Ae. albopictus* initially invades a new habitat. The left (**e**) shows the tire scenario with competition parameters favoring *Ae. albopictus* ($\alpha _t = 0.25 < \alpha _a = 0.83$). The right (**f**) shows the competition parameters that favor *Ae. triseriatus* ($\alpha _t = 0.73 > 0.42 =\alpha _a$). All parameters are chosen to be the values in Table 1 with all *Ae. albopictus* parasite parameters set to 1

To begin, we model the population dynamics of *Ae. albopictus* and *Ae. triseriatus* without parasitism. We consider two separate environments: tire and treehole. The difference between the two scenarios is the Lokta-Volterra competition parameters ($\alpha _t$ and $\alpha _a$) that were fit for each environment from Livdahl and Willey [16]. In both cases, the populations settle to an equilibrium. In the tire environment, the population ends in category 6, in which *Ae. albopictus* dominates, but *Ae. triseriatus* remains at low levels (Fig. 2, right). After 150 days, we find 126 adult *Ae. albopictus* compared to about 5 adult *Ae. triseriatus*. For the treehole environment, the population ends in category 5 (Fig. 2, left), where *Ae. albopictus* is still the dominant species, but *Ae. triseriatus* has a sizeable population. After 150 days, there are approximately 54 *Ae. albopictus* and 19 *Ae. triseriatus*. Parameter values are the estimated averages from the literature and are found in Table 1.

To account for known variability in parameters, we examine dynamics across a broad parameter space using a LHS with the ranges indicated in Table 1. Assuming no effects of parasitism in *Ae. albopictus* and a low effect in *Ae. triseriatus*, we find that the majority, 56%, of simulations fall into category 6 (Fig. 2a), where *Ae. albopictus* strongly dominates. This was the same result as in the tire scenario. In addition, approximately 25% of the simulations fall into the same category as the treehole scenario (category 5). Only 3.5% of the total 100,000 samples lead to categories in which *Ae. triseriatus* has a larger population than *Ae. albopictus* (categories 1, 2, and 3). A further 10.5% of the simulations fall into coexistence with approximately equal amounts of both species (category 4). For a small number of simulations (5%), *Ae. albopictus* completely eliminated *Ae. triseriatus* without parasitism (category 7), but the most common scenario was for *Ae. albopictus* to be the dominant species and strongly limit the population of *Ae. triseriatus*.

**Table 3:** Dilution parasitism parameters. Each level variation for parasitism (high or low) for each species

*Ae. albopictus*	max $\gamma _{m_a}$	max $\gamma _{d_a}$	max $\gamma _{b_a}$	*Ae. triseriatus*	max $\gamma _{m_t}$	max $\gamma _{d_t}$
Low	1.5	1.2	1.2	Low	1.5	1.2
High	16	4	4	Low	1.5	1.2
Low	1.5	1.2	1.2	High	8	4
High	16	4	4	High	8	4

If we increase the effect of parasitism in *Ae. triseriatus*, we find that there is a strong shift in scenarios to where *Ae. albopictus* is the dominant species (Fig. 2c). In this case, approximately 66% of parameters selected fall into category 6 (few *Ae. triseriatus*) and 9.9% of situations lead to elimination of *Ae. triseriatus* (category 7). *Ae. triseriatus* only has a greater proportion than *Ae. albopictus* in approximately 1% of the parameters sampled.

**Fig. 3:**
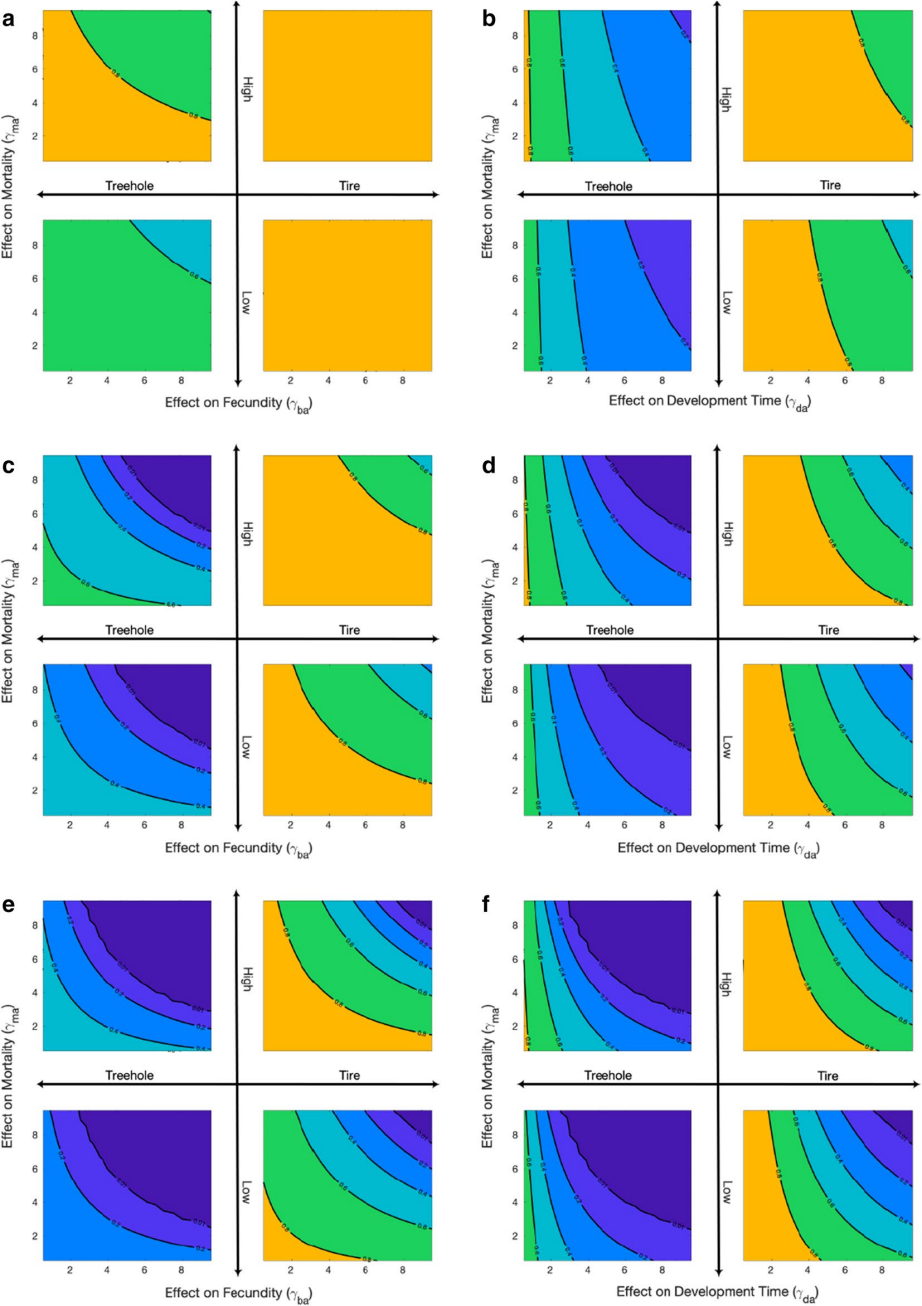
Effects of parasitism on *Ae. albopictus* proportions. The proportion of *Ae. albopictus* when varying parasitism parameters. For all subplots, the right quadrants (I and IV) are the tire environment where there is a greater competitive effect on *Ae. triseriatus* than on *Ae. albopictus*; the left quadrants (II and III) are the treehole environment where the reverse is true; the upper quadrants (I and II) have high parasite effects on *Ae. triseriatus*; the lower quadrants (III and IV) have a low parasite effect on *Ae. triseriatus*. All three *Ae. albopictus* parasitism parameters—$\gamma _{b_a}$, $\gamma _{m_a}$, and $\gamma _{d_a}$—are varied. All subplots vary mortality on the y-axis while the left subplots (**a**, **c**, **e**) have fecundity on the x-axis, and the right subplots (**b**, **d**, **f**) have development time on the x-axis. Each row fixes the third parasite parameter as not varied: 1 (**a**, **b**), 3 (**c**, **d**), 5 (**e**, **f**). Lines distinguish between different outcome categories. For example, yellow is when the proportion of *Ae. albopictus* exceeds 0.8

### Including parasitism in *Ae. albopictus*

When we include the effects of parasitism for *Ae. albopictus* in the dynamics, we greatly increase the parameter space where *Ae. triseriatus* is the dominant species. We use LHS but include the parameters for parasitism: $\gamma _{d_a}$, $\gamma _{m_a}$, and $\gamma _{b_a}$. We find that results falling in categories 1–4 have greatly increased while those in categories 6 and 7 have significantly decreased (Fig. 2 b, d). In fact, *Ae. triseriatus* completely eliminates (category 1) *Ae. albopictus* in approximately 15.0% and 15.3% of the simulations for low and high *Ae. triseriatus* parasitism, respectively. However, in 30.7% and 24.4% of the simulations *Ae. triseriatus* is the dominant species (categories 1, 2, 3), and the sum of all categories where *Ae. albopictus* will be the dominant species is reduced to 54.7% and 64.8% (sum of categories 5, 6, 7) with low and high parasitism for *Ae. triseriatus*, respectively. The total simulations with outcomes in categories 5, 6, and 7 are similar to the amount in only category 6 without *Ae. albopictus* parasitism. With parasitism of *Ae. albopictus*, category 6 still has the most outcomes, but the outcomes spread out. Overall, we see that without parasitism *Ae. albopictus* will be the dominant vector; however, once parasitism takes place *Ae. triseriatus* can become dominant.

**Fig. 4:**
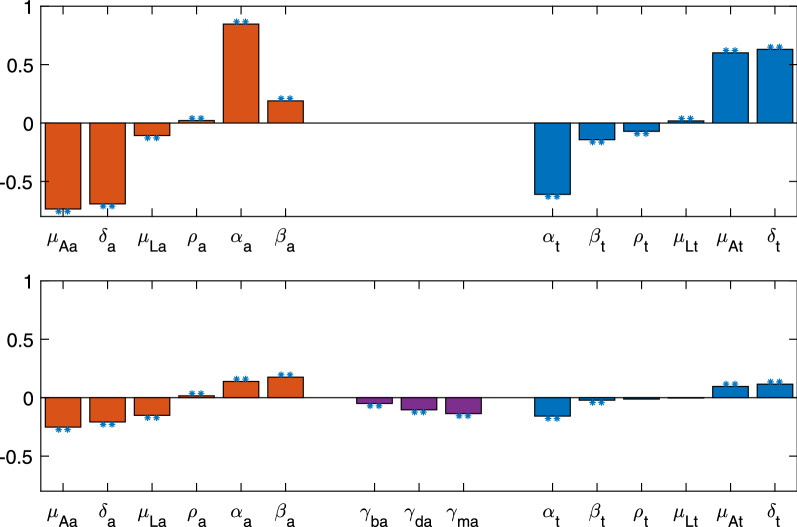
Partial rank correlation coefficients of the parameter values with the final proportion of *Ae. albopictus* at 2000 days. The stars indicate significance with a *p*-value < 0.00001. The top plot shows the PRCC with the LHS with all parasitism parameters set to 1 ($\gamma _{d_a} =\gamma _{m_a} =\gamma _{b_a} =1$), indicating no parasitism. The bottom plot is the PRCC with variation of parasitism included in the LHS for *Ae. albopictus*. Parasitism on *Ae. triseriatus* is low. Red bars (left side) are parameters associated with *Ae. albopictus*, and blue bars (right side) with *Ae. triseriatus*. Parasitism parameters on *Ae. albopictus*, which only occur in the bottom plot, are shown by purple bars (middle). For each grouping, parameters are ordered from the least to greatest effect when including parasitism. The PRCC for parameter values with high *Ae. triseriatus* parasitism is found in Additional file 1: Figure S5

To further examine the effects of parasitism, we vary the level of each of the three parasitism parameters. We perform these variations pairwise under four environmental scenarios: tire (Fig. 3, quadrants I and IV) and tree hole (Fig. 3, quadrants II and III) with both a low and high parasite effect on *Ae. triseriatus*. Recall the difference between the two scenarios is the competition parameters ($\alpha _t$ and $\alpha _a$). In the tire scenario, the competition effect of *Ae. albopictus* on *Ae. triseriatus* is greater ($\alpha _a = 0.83 > 0.25 = \alpha _t$), and for the treehole scenario, the competition effect of *Ae. triseriatus* on *Ae. albopictus* is greater ($\alpha _a = 0.42 < 0.73 = \alpha _t$). All other parameters are fixed (Table 1). The colors in these images correspond to the colors of the seven categories from Table 2, although only six colors appear as category 7 never occurs in these simulations. Parasitism effects were varied pairwise. First, parasite effects on larval mortality ($\gamma _{m_a}$) and larval development time ($\gamma _{d_a}$) were varied with three constant levels of the effect on female fecundity ($\gamma _{b_a} = 1,3,5$) (Fig. 3). Then, parasite effects on female fecundity ($\gamma _{b_a}$) and larval development time ($\gamma _{d_a}$) were varied with three constant levels of the effect on mortality ($\gamma _{m_a} = 1,3,5$). Finally, parasite effects on larval mortality ($\gamma _{m_a}$) and female fecundity ($\gamma _{b_a}$) were varied with three constant levels of the effect on larval development time ($\gamma _{m_a} = 1,3,5$). We present these results in Additional file 2 as they are very similar to those found from fecundity versus development time.

**Fig. 5:**
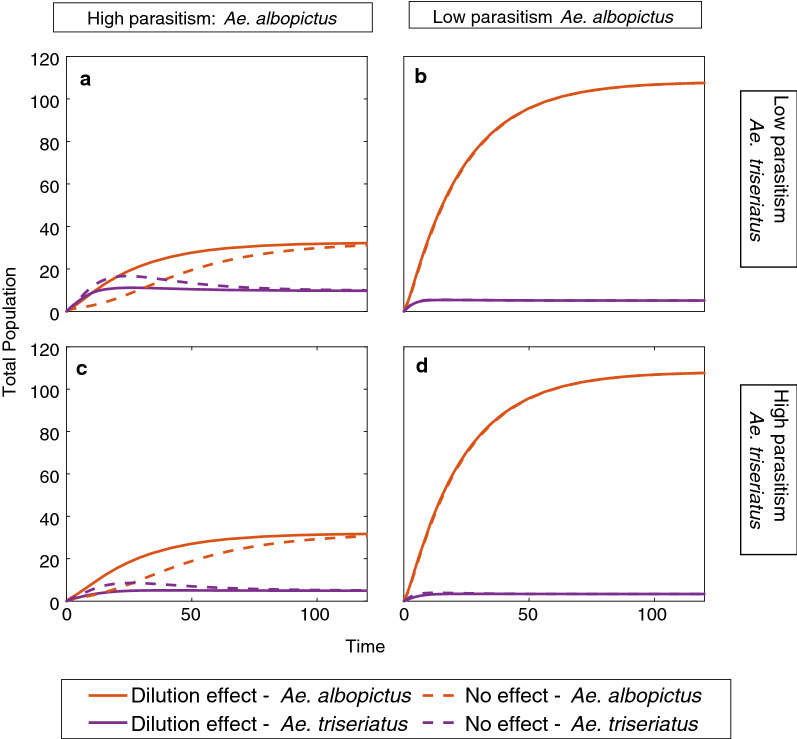
Dilution effects of parasitism. The proportion of *Ae. albopictus* when varying the parasitism parameters. All subplots are in the tire scenarios ($\alpha _a = 0.83$ and $\alpha _t = 0.25$), where the effect of competition on *Ae. triseriatus* is greater than on *Ae. albopictus*. The solid lines indicate dilution of parasitism, which means that parasite parameters of a species decrease as their proportion decreases. The dashed lines are in the absence of dilution and assume a constant parasite parameter. See the text for how the values are chosen. The left (**a**, **c**) shows the maximum parasite parameters are larger for *Ae. albopictus*, and the right (**b**, **d**) has smaller maximum parasite parameters. The top (**a**, **b**) shows smaller maximum parasite parameters for *Ae. triseriatus* and the bottom (**c**, **d**) larger values. All parameters are listed in Table 3

In the tire scenario ($\alpha _a = 0.83 > 0.25 = \alpha _t$), we find that in large portions of the parameter space *Ae. albopictus* strongly dominates, i.e. category 6 (Fig. 3, quadrants I and IV, dark yellow). When the effect on female fecundity ($\gamma _{b_a}$) is fixed at 1, the transition to a greater level of coexistence between the mosquito species is only seen at higher levels of effects on the larval development rate ($\gamma _{d_a}$), with values around $\gamma _{d_a}> 4$ (Fig. 3b, quadrant I and IV). For female fecundity ($\gamma _{b_a}$) fixed at three and five (Fig. 3d, f, quadrant I and IV), development time effects as low as $\gamma _{d_a} \approx 2$ result in a shift to a low proportion of *Ae. albopictus* (category 5 green). For *Ae. triseriatus* to be the dominant species, there must be very high effects of all three parasitism parameters affecting *Ae. albopictus*, regardless of *Ae. triseriatus*’ level of parasitism.

Parasite effects on the development time have more significant effects than on mortality or fecundity. In the tire scenario, this is most apparent when there is no change in the development time (Fig. 3a); then, *Ae. albopictus* always strongly dominates. Additionally, there is no change in category, regardless of the strength of the effect on mortality and fecundity or parasitism on *Ae. triseriatus*. In the tire scenario, if the effect on development time is increased by three times (Fig. 3c), effects on both fecundity and mortality must be relatively high to obtain higher levels of *Ae. triseriatus*. Furthermore, *Ae. triseriatus* will only dominate if parasite effects on both fecundity and mortality are > 10. When the development time in tires is five times greater, only a small effect by the other two parameters is needed to decrease the proportion of *Ae. albopictus* to category 5. Similarly, with fixed fecundity, when the parasite increases the development time seven fold, we see that regardless of the other two parameters, the proportion of *Ae. albopictus* decreases to a minimum of category 5 (Fig. 3f, green and blue).

In the treehole scenario, *Ae. triseriatus* has a more competitive effect on *Ae. albopictus*. As seen with the absence of parasitism for *Ae. albopictus* with a low effect of parasitism on *Ae. triseriatus*, the default is category 5 (Fig. 3a, b, quadrant III, green in bottom left corner), in which *Ae. albopictus* is still the dominant species, but there are more *Ae. triseriatus*. In this situation, the effect on development time must only increase two-fold for the proportion of *Ae. albopictus* to decrease to category 4 (Fig. 3b, quadrant III, teal), in which there are relatively similar amounts of both species. When the effect on fecundity is set to 3 (Fig. 3d, quadrants II, III) and the effect of development time is > 4, *Ae. triseriatus* will be the dominant species (all three shades of blue). When the effect of fecundity is set to 5 (Fig. 3f, quadrants II, III) and the development time is > 3, *Ae. triseriatus* will dominate. A similar result is seen when the development time is five times greater (Fig. 3e, quadrants II, III). For all values of the other two parameters, *Ae. triseriatus* is the dominant species. In fact, almost half of the parameter space considered falls into category 1 where *Ae. albopictus* is eliminated (darkest blue). Without any parasite effects on development time (Fig. 3a, quadrants II, III), there is only a chance for the two species to be relatively equal if both other parameters are > 7 and there is a low effect of parasitism on *Ae. triseriatus*.

Overall, in tires, only with very high effects on all three parameters do we find that *Ae. albopictus* is dominated by *Ae. triseriatus*, while in treeholes, we find that less parasitism is needed to eliminate *Ae. albopictus*, i.e. only high effects on two of the *Ae. albopictus* parasite parameters. In both scenarios, as we increase the effects of the parasite parameters we see that the proportion of *Ae. albopictus* decreases. The change is most notable as the parasite effect on *Ae. albopictus* development time is increased, regardless of the scenario.

### Importance of parameters

From the parameters selected using LHS with a low effect of parasitism on *Ae. triseriatus*, we looked at the partial rank correlation coefficient (PRCC) for each parameter with respect to the final proportion of *Ae. albopictus* at 2000 days. In the case without parasitism, we find that all parameters have a significant impact on the model (Fig. 4, top row). When parasitism is included (Fig. 4, bottom row), the parameters which are not significant are all related to *Ae. triseriatus*: death of larvae, $\mu _{L_t}$, and proportion of females, $\rho _t$.

In the case without parasitism, the parameter with the most influence (largest PRCC value in magnitude) is the competition parameter $\alpha _a$, which is the inter-specific competition effect on *Ae. triseriatus* from *Ae. albopictus*. With a PRCC value of 0.8469, the more $\alpha _a$ increases, the greater the proportion of *Ae. albopictus* is. Several parameters have a fairly large correlation. The following parameters all have a PRCC value > 0.5 in magnitude (in the order of the greatest magnitude to lowest): death rate of adult *Ae. albopictus*, $\mu _{A_a}$; *Ae. albopictus* larval development time, $\delta _a$; *Ae. triseriatus* larval development time, $\delta _t$, the competition parameter $\alpha _t$ on *Ae. albopictus*; and death rate of adult *Ae. triseriatus*, $\mu _{A_t}$. We see that the most important parameters are the death rates of adults, transition rate to adults, and Lokta-Volterra competition parameters.

When we introduce the parasitism parameters into the LHS, we see that the parasite’s effect on development time $\gamma _{m_a}$ has the sixth greatest impact, but the largest of all the parasitism parameters. With a PRCC of $-0.1359$, we expect the increase of $\gamma _{m_a}$ to decrease the proportion of *Ae. albopictus*. The death of adult *Ae. albopictus*, *Ae. albopictus* larval development time $\delta _a$, *Ae. albopictus* birth rate $\beta _a$, Lokta-Volterra competition parameter $\alpha _t$, and death of *Ae. albopictus* larvae all have a magnitude of PRCC values greater than $\gamma _{d_a}$ (− 0.2525, − 0.2081, 0.1754, − 0.1517, and − 0.1581, respectively). While less important than $\gamma _{m_a}$, the other two parasite parameters are statistically significant ($p<0.00001$) as well. The PRCC value for the parasite’s effect on development time is $-0.1039$, and for the parasite’s effect on fecundity is $-0.05$.

When we consider the LHS with a high effect of parasitism on *Ae. triseriatus*, all parameters have a much lower effect, as indicated by the lower magnitude of the PRCC value. The parameter with the greatest magnitude PRCC (0.1666) is the Lokta-Volterra competition parameter $\alpha _a$; this is the same parameter that was found to have the most impact when considering a low effect of parasitism on *Ae. triseriatus* and no parasite effect on *Ae. albopictus*. In general, the trends of PRCC values for the parameters are similar to those with a low effect of parasitism on *Ae. triseriatus*. See Additional file 1: Figure S5.

### Dilution effects

When we consider including dilution effects into parasitism, we find that the equilibrium values appear to be identical to the case without dilution. To ensure comparison of equivalent cases, we find the equilibrium proportion $\hat{P}_a$ of *Ae. albopictus* larvae under dilution. We use this in our dilution formula $\gamma _i = (\gamma _{max} -1)\hat{P}_a + 1$ to find comparable parasitism parameters for the absence of dilution. Thus, at our starting condition the parasite parameters in the two cases differ because of the different population proportions, but approach the same values at equilibrium (Fig. 5). In Fig. 5, we plotted four different situations in which we have either a high or low parasite effect for the initial maximum parasite value (Table 3). While the equilibria are identical, there is a difference in the dynamics before reaching equilibrium. This is particularly apparent in the case where *Ae. albopictus* starts with a high parasite effect. In this case, *Ae. triseriatus* initially has a greater population before *Ae. albopictus* becomes the dominant species.

### Analytical formulation of the proportion of *Ae. albopictus*

We analyzed our model to determine the long-term behavior for the proportion of *Ae. albopictus*. From the system of equations, there are four possible equilibria: extinction of mosquitoes, competitive exclusion with each species type present, and coexistence. The equilibrium equations can be found in the Additional file 3. Before we discuss our formula for the population proportion, we introduce the ratio which represents the reproduction number of each species, $R_t$ and $R_a$, for *Ae. triseriatus* and *Ae. albopictus*, respectively. These are given by:

$$\begin{aligned} R_a= & {} \displaystyle \frac{\beta _a\rho _a}{\gamma _{b_a}}~\frac{1}{\mu _{A_a}}~\frac{\frac{1}{\gamma _{d_a}\delta _a}}{\frac{1}{\gamma _{d_a}\delta _a} + \gamma _{m_a}\mu _{L_a}},\\ R_t= & {} \displaystyle \beta _t\rho _t ~\frac{1}{\mu _{A_t}}~\frac{\frac{1}{\gamma _{d_t}\delta _t}}{\frac{1}{\gamma _{d_t}\delta _t} + \gamma _{m_t}\mu _{L_t}}. \end{aligned}$$

In each reproduction number, the first term is the birth rate, $\frac{\beta _a\rho _a}{\gamma _{b_a}},$ for *Ae. albopictus* and $\beta _t \rho _t$ for *Ae. triseriatus*. This is multiplied by the inverse of the adult death rate, i.e. the expected life span of an adult mosquito. Together, these terms are the total expected number of eggs a female will lay in her lifetime. The final term has the development rate (i.e. $\frac{1}{\gamma _{d_t}\delta _t}$ and $\frac{1}{\gamma _{d_a}\delta _a}$, respectively), divided by the sum of the development rate and death rate of larvae. This proportion is the probability of an egg surviving to adulthood. Overall, the reproductive number gives the total surviving children that a single female will lay. If $R_t> 1$, the population of *Ae. triseriatus* can establish. Similarly, if $R_a>1$, *Ae. albopictus* can establish.

We determine the equilibrium for coexistence in terms of $R_t$ and $R_a$ (see Additional file 3). From these, we calculate the equilibrium proportion of adult *Ae. albopictus* by:

$$\begin{aligned} \frac{A_a}{A_a+A_t} = \displaystyle \frac{w_d\bigl (1-\alpha _t + \frac{\alpha _t }{R_t}- \frac{1}{R_a}\bigr )}{w_d\bigl (1-\alpha _t + \frac{\alpha _t }{R_t}- \frac{1}{R_a}\bigr ) + q_d\bigl (1-\alpha _a + \frac{\alpha _a }{R_a}- \frac{1}{R_t}\bigr )} \end{aligned}$$

where

$$\begin{aligned} w_d= & {} \frac{1}{\gamma _{d_a}\delta _a\mu _{A_a}}, \\ q_d= & {} \frac{1}{\gamma _{d_t}\delta _t\mu _{A_t}}. \end{aligned}$$

Notice that the parameters $w_d$ and $q_d$ are the inverse of the quantity of development time multiplied by the adult death rate for *Ae. albopictus* and *Ae. triseriatus*, respectively. This represents the expected life span. From our analytical description of the proportion of *Ae. albopictus*, we can determine the importance of different parameters.

## Discussion

While some studies suggest that *Ae. albopictus* is the stronger competitor, consistently eggs of both species are found together in the wild even in the presence of high levels of parasitism [3, 7, 23, 55]. Indeed, our results show that high levels of infection of *Ae. albopictus* with *As. taiwanensis* have a significant effect on the population levels and level the playing field between the two mosquito species. We find a wide range of situations in which *Ae. triseriatus* is the dominant species, but primarily in the presence of unrealistically high effects of parasitism in *Ae. albopictus* (Fig. 3). Thus, it is unlikely that *Ae. triseriatus* would dominate *Ae. albopictus* without significant drastic effects of parasitism on the mosquito species. Furthermore, the combined effects of the parasite would likely not result in complete elimination of *Ae. albopictus* in the wild.

There are additional factors other than the effects of *gregarine* parasites, that contribute to coexistence. One of these is that the competitive effects are lessened when resources are sufficient [17]. Although *Ae. triseriatus* develops more slowly and has a smaller survival rate, it can survive at a lower temperature than *Ae. albopictus* [18]. We also make the assumption that the reproductive cycles and seasons of the two *Aedes* mosquitoes overlap completely. However, the populations of the two mosquitoes peak at different times: *Ae. triseriatus* peaks in late June and early July, while *Ae. albopictus* peaks in late July through late August [3], which could give *Ae. triseriatus* a slight edge that is not incorporated into this model. Although this is most likely due to other factors, we also saw these dynamics when we considered the dilution of parameters and that *Ae. albopictus* has a high parasite effect. These temporal changes where *Ae. triseriatus* initially has a greater population, but then returns to the same coexistence equilibrium, show that the initial seasonal population of *Ae. triseriatus* might return to the same equilibrium with the return of *Ae. albopictus*. It would be possible to study these alterations by changing the initial conditions of the simulations, for example, changing the initial conditions so that *Ae. triseriatus* starts with both adults and larvae and *Ae. albopictus* starts only with larvae. Additionally, we could simulate a later introduction of *Ae. albopictus* to compensate for this difference.

*Ae. triseriatus* is native to the areas in which it competes with *Ae. albopictus* and is subsequently unable to escape its parasite in the same way as *Ae. albopictus*. Consequently, a fixed level parasitism is assumed to occur when the population is at equilibrium. We only consider two levels of infection of *Ae. triseriatus* by its parasite *As. barretti*, which affects the development time and mortality of *Ae. triseriatus*. Thus, we do not consider all possible combinations of parasitism, but it does indicate that the increase of parasitism on *Ae. triseriatus* increased the proportion of *Ae. albopictus*, but did not drastically affect the overall dynamics. Additional evidence suggests that *Ae. triseriatus* that are infected with *As. barretti* were killed less often by a predator, *Toxohrynchites rutilus*, compared to uninfected *Ae. triseriatus* [56]. This shows that while the effect of the parasite does have an effect on the competition between the two species, there are other species that might exacerbate or mitigate the effects of *As. taiwanensis* on *Ae. albopictus*.

Evidence suggesting that endemic infection of *Ae. albopictus* by *As. taiwanensis* decreases fitness has implications for the spread of mosquito-borne diseases. In southwest Virginia, both *Aedes* species can act as a vector for La Crosse encephalitis virus (LACV). While *Ae. triseriatus* is the primary vector for the virus, evidence suggests that as *Ae. albopictus* becomes more established, it can transmit LACV just as effectively as *Ae. triseriatus* [11, 12]. Importantly, as *Ae. albopictus* often occurs in urban or para-urban settings, it can act as a bridge vector for LACV to the human population. While La Crosse encephalitis is not widespread across the US, it is one of the most common mosquito-borne pediatric diseases in the country [57]. LACV infections result in inflammation of the brain, which can lead to seizures and paralysis [2]. In the future, this understanding of mosquito population dynamics can be implemented in the study of mosquito-borne diseases such as La Crosse encephalitis.

## Conclusion

We aimed to determine the extent to which parasitism of *Ae. albopictus* by *As. taiwanensis* impacts its competition with *Ae. triseriatus*. Both *Aedes* mosquitoes are potentially competent vectors for a number of human arboviruses, and insight into the dynamics of these two species could help inform future disease mitigation efforts. Without parasitism due to *As. taiwanensis*, *Ae. albopictus* has a large and distinct competitive advantage over *Ae. triseriatus* in some environments. As *Ae. albopictus* is an often invasive generalist species, its domination over native *Ae. triseriatus* in the absence of mitigating effects, such as parasitism, is likely. Our results suggest that the competitive advantage of *Ae. albopictus*, in the absence of parasitism, is so great that if the species has even the slightest edge over *Ae. triseriatus*, it will dominate given enough time. From our parameter sweep, 86% of the scenarios resulted in *Ae. albopictus* being the dominant species, with few scenarios leading to *Ae. triseriatus* as the dominant species, under low parasitism on *Ae. triseriatus*. When we increase the effect of parasitism of *Ae. triseriatus* to a high level, *Ae. albopictus* is dominant in 94% of the scenarios. Even in treeholes where the competition effect from *Ae. triseriatus* is greater, *Ae. albopictus* remains the dominant species. This is consistent with previous results [7, 16, 23]. When considering the impact of parasitism of *Ae. albopictus* by *As. taiwanensis*, we observe a decreased overall fitness of the species and negative effects on its competitive ability. Previous empirical results corroborate our findings [7]. To be clear, the reduction of individual traits is assumed, but our results indicate that the overall population levels are significantly affected by changes in these individual traits. This is seen when changing the effect of parasitism (Fig. 3). The three parasitism parameters all have significant effects on the balance between the two species, with the parasite effect on development time having the strongest effect on the proportion of *Ae. albopictus*. The greatest effect is observed when there is a combined effect on female fecundity, larval development time, and larval mortality. As we increase the effects of the parasite parameters we see that the proportion of *Ae. albopictus* decreases and leads to elimination of *Ae. albopictus* when parasitism is extremely high.

## Supplementary Information

**Additional file 1.** Equilibrium and supplementary figure.

**Additional file 2.** Code.

**Additional file 3.** Generated data.

## Data Availability

The datasets generated during the current study are available in its additional files.

## Abbreviations

Ae.: *Aedes*
LACV: La Crosse encephalitis virus
PRCC: partial regression correlation coefficients
As.: *Ascogregarina*
GRR: Gross reproductive rate
GC: Gonotrophic cycle
