# Supplementary material for: Parasitism of Aedes albopictus by Ascogregarina taiwanensis lowers its competitive ability against Aedes triseriatus
Source: Parasit Vectors. 2021 Jan 25;14:79. doi: 10.1186/s13071-021-04581-0 (PMC7831270; doi:10.1186/s13071-021-04581-0)
Supplement: Supplementary file 1 — Additional file 1. Equilibrium and supplementary figure. [file 13071_2021_4581_MOESM1_ESM.pdf]

Additional file 1 — Equilibrium and Supplemental Figures

There are four possible equilibrium ($L_a^*,\ A_a^*,\ L_t^*,\ A_t^*$) for our system of equations as described below.

1. Infection free equilibrium
$$(0, 0, 0, 0).$$

2. Competitive exclusion with only *Ae. triseriatus*
$$\left(0, 0, \hat{L}_t, q_d\hat{L}_t\right),$$
where
$$\begin{aligned}
\hat{L}_t &= K\left(1 - \frac{1}{R_t}\right), \\
R_t &= \frac{1}{\gamma_{d_t}\delta_t\left(\frac{1}{\gamma_{d_t}\delta_t} + \gamma_{m_t}\mu_{L_t}\right)}\frac{\beta_t\rho_t}{\mu_{A_t}}, \\
q_d &= \frac{1}{\gamma_{d_t}\delta_t\mu_{A_t}}.
\end{aligned}$$

3. Competitive exclusion with only *Ae. albopictus*
$$\left(\hat{L}_a, w_d\hat{L}_a, 0, 0\right),$$
where
$$\begin{aligned}
\hat{L}_a &= K\left(1 - \frac{1}{R_a}\right), \\
R_a &= \frac{1}{\gamma_{d_a}\delta_a\left(\frac{1}{\gamma_{d_a}\delta_a} + \gamma_{m_a}\mu_{L_a}\right)}\frac{\beta_a\rho_a/\gamma_{b_a}}{\mu_{A_a}}, \\
w_d &= \frac{1}{\gamma_{d_a}\delta_a\mu_{A_a}}.
\end{aligned}$$

4. Coexistence of both species
$$\left(\tilde{L}_a, w_d\tilde{L}_a, \tilde{L}_t, q_d\tilde{L}_t\right),$$
where
$$\begin{aligned}
\tilde{L}_a &= \frac{K}{1 - \alpha_t\alpha_a}\left(1 - \alpha_t + \frac{\alpha_t}{R_t} - \frac{1}{R_a}\right), \\
\tilde{L}_t &= \frac{K}{1 - \alpha_t\alpha_a}\left(1 - \alpha_a + \frac{\alpha_a}{R_a} - \frac{1}{R_t}\right).
\end{aligned}$$

The main text gives a description of the biological meaning of the population reproduction numbers R_t and R_a.

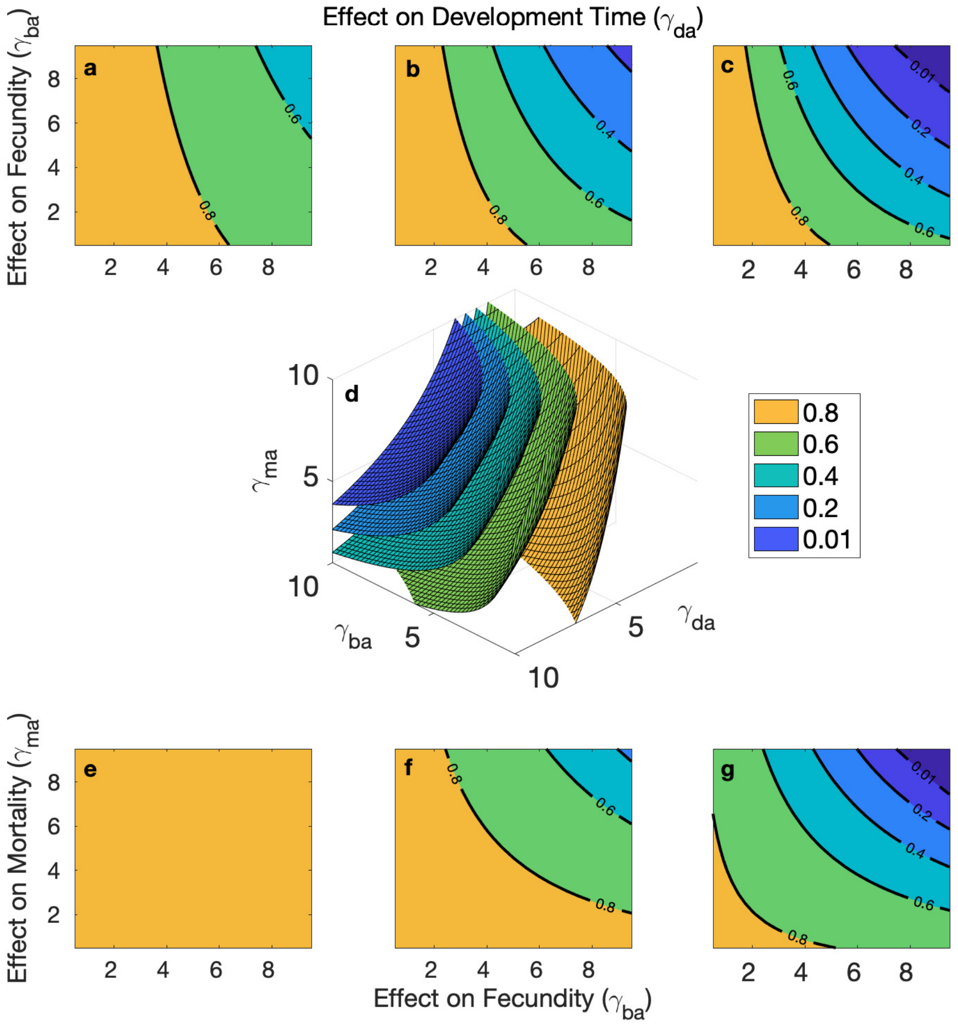

Figure 1: Effects of parasitism on *Ae. albopictus* in tires with low parasitism on *Ae. triseriatus*. This shows the proportion of *Ae. albopictus* when varying *Ae. albopictus* parasitism parameters. All figures have competition parameters $\alpha_a = 0.83$ and $\alpha_t = 0.25$, which means that competition is in favor of *Ae. albopictus*. All three parasitism parameters – γ_{b_a}, γ_{m_a}, γ_{d_a} – are varied. Across row one and three, the parasitism parameter not being varied (i.e. not on the x or y-axis) is fixed at 1 (a,e), 3 (b,f), or 5 (c,g). The first row (a,b,c) varies the parasites effect on development time (x-axis) and fecundity (y-axis) with the parasites effect on mortality changing from 1, 3 to 5 from left to right. The third row (e,f,g) show effects of fecundity (x-axis) and effects of mortality (y-axis) with effects on development time being 1,3, and 5 from left to right. The figure in the second row is a three dimensional depiction of the contour lines with effects on mortality (z-axis), on development time (y-axis) and on fecundity (x-axis). Notice that the effect on development time (y-axis) has been rotated so that the it goes from largest to smallest. Lines distinguish between different outcome categories. For example, yellow is when the proportion of *Ae. albopictus* exceeds 0.8.

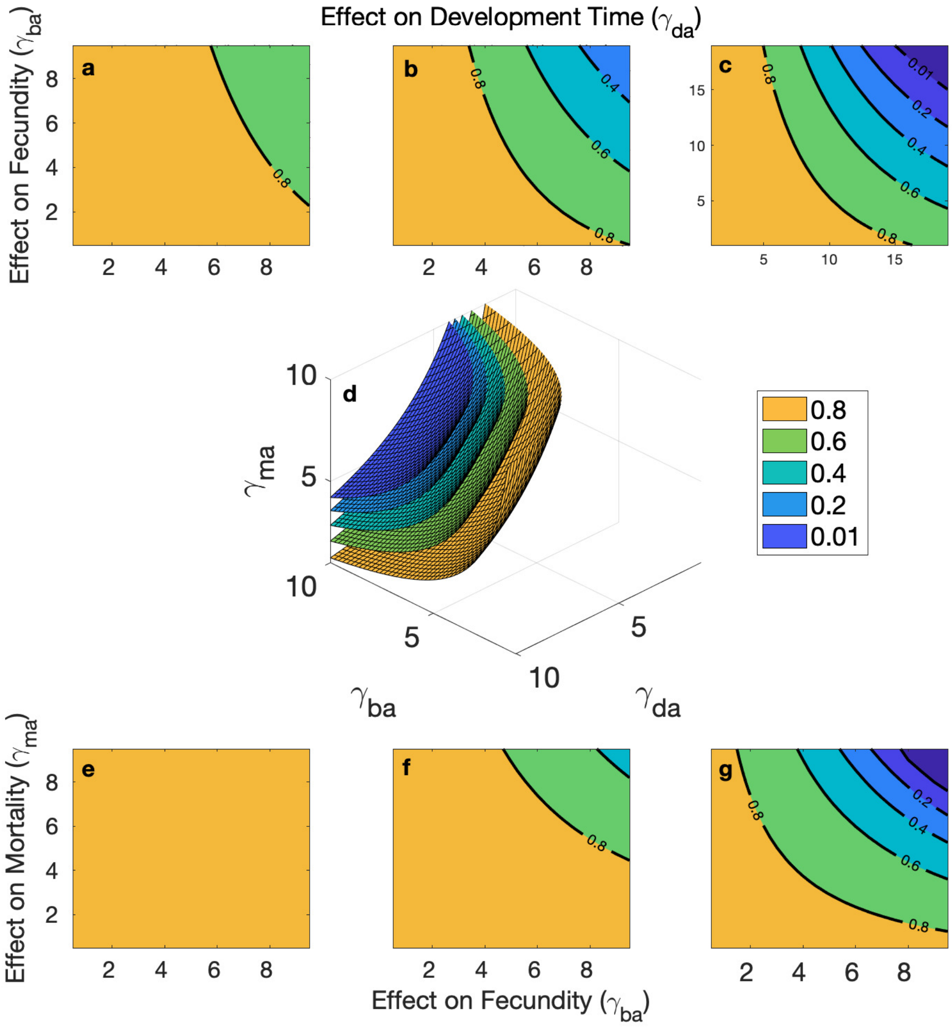

Figure 2: Effects of parasitism on *Ae. albopictus* in tires with high parasitism on *Ae. triseriatus*. This shows the proportion of *Ae. albopictus* when varying *Ae. albopictus* parasitism parameters. All figures have competition parameters $\alpha_a = 0.83$ and $\alpha_t = 0.25$, which means that competition is in favor of *Ae. albopictus*. All three parasitism parameters – γ_{b_a}, γ_{m_a}, γ_{d_a} – are varied. Across row one and three, the parasitism parameter not being varied (i.e. not on the x or y-axis) is fixed at 1 (a,e), 3 (b,f), or 5 (c,g). The first row (a,b,c) varies the parasites effect on development time (x-axis) and fecundity (y-axis) with the parasites effect on mortality changing from 1, 3 to 5 from left to right. The third row (e,f,g) show effects of fecundity (x-axis) and effects of mortality (y-axis) with effects on development time being 1,3, and 5 from left to right. The figure in the second row is a three dimensional depiction of the contour lines with effects on mortality (z-axis), on development time (y-axis) and on fecundity (x-axis). Notice that the effect on development time (y-axis) has been rotated so that the it goes from largest to smallest. Lines distinguish between different outcome categories. For example, yellow is when the proportion of *Ae. albopictus* exceeds 0.8.

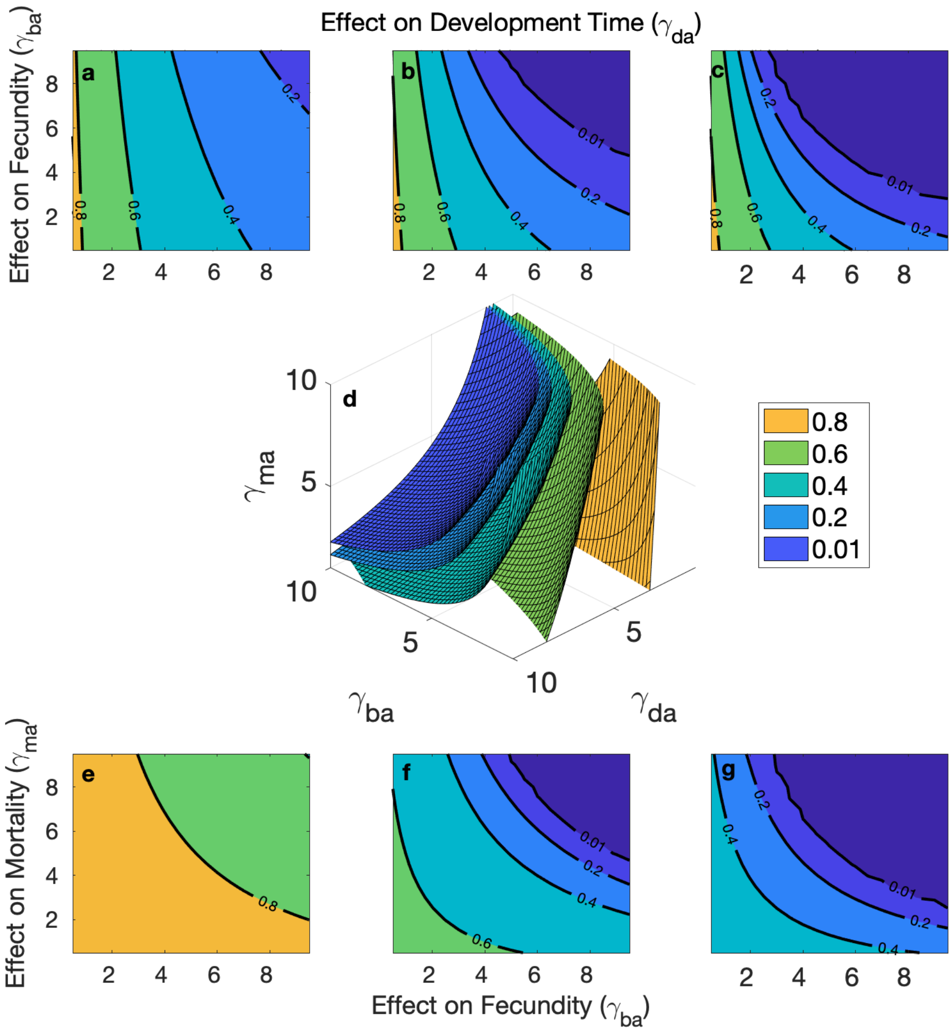

Figure 3: Effects of parasitism on *Ae. albopictus* in tires with high parasitism on *Ae. triseriatus*. This shows the proportion of *Ae. albopictus* when varying *Ae. albopictus* parasitism parameters. All figures have competition parameters $\alpha_a = 0.42$ and $\alpha_t = 0.73$, which means that competition is in favor of *Ae. triseriatus*. All three parasitism parameters – γ_{b_a}, γ_{m_a}, γ_{d_a} – are varied. Across row one and three, the parasitism parameter not being varied (i.e. not on the x or y-axis) is fixed at 1 (a,e), 3 (b,f), or 5 (c,g). The first row (a,b,c) varies the parasites effect on development time (x-axis) and fecundity (y-axis) with the parasites effect on mortality changing from 1, 3 to 5 from left to right. The third row (e,f,g) show effects of fecundity (x-axis) and effects of mortality (y-axis) with effects on development time being 1,3, and 5 from left to right. The figure in the second row is a three dimensional depiction of the contour lines with effects on mortality (z-axis), on development time (y-axis) and on fecundity (x-axis). Notice that the effect on development time (y-axis) has been rotated so that the it goes from largest to smallest. Lines distinguish between different outcome categories. For example, yellow is when the proportion of *Ae. albopictus* exceeds 0.8.

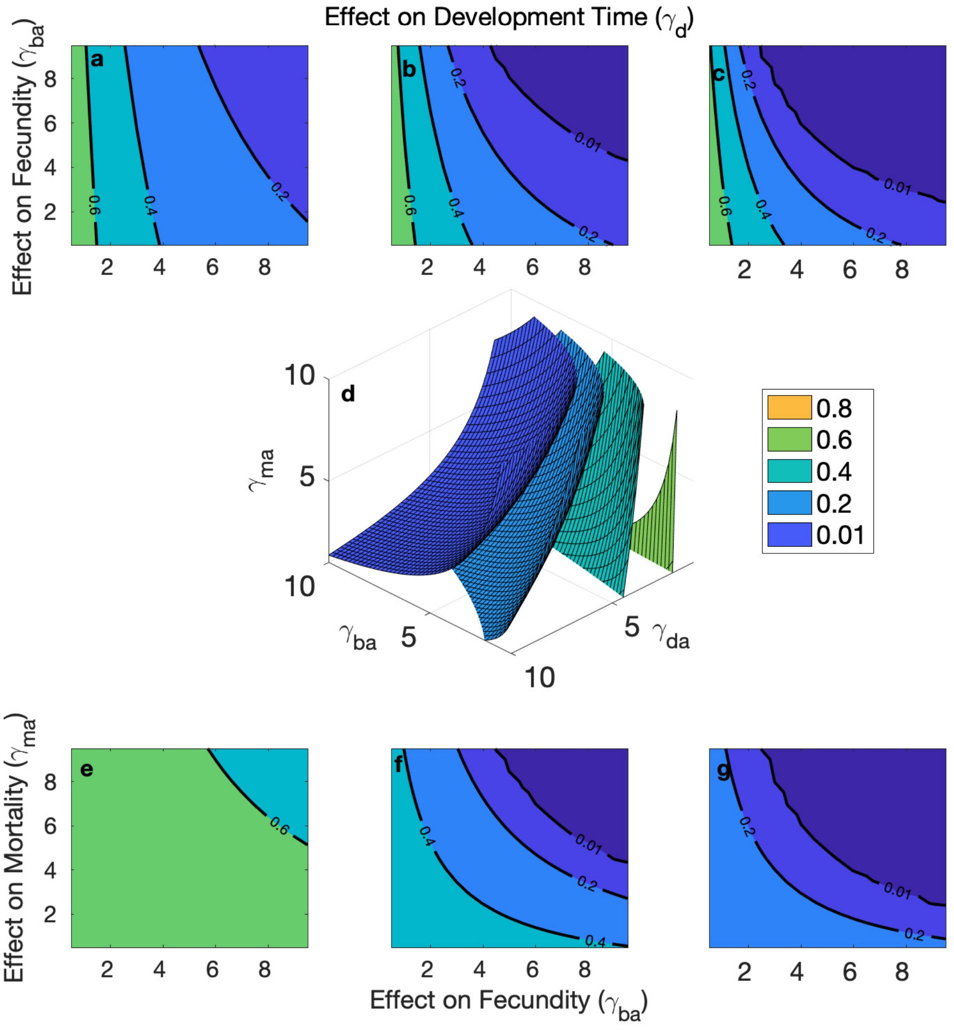

Figure 4: Effects of parasitism on *Ae. albopictus* in tires with low parasitism on *Ae. triseriatus*. This shows the proportion of *Ae. albopictus* when varying *Ae. albopictus* parasitism parameters. All figures have competition parameters $\alpha_a = 0.42$ and $\alpha_t = 0.73$, which means that competition is in favor of *Ae. triseriatus*. All three parasitism parameters – γ_{b_a}, γ_{m_a}, γ_{d_a} – are varied. Across row one and three, the parasitism parameter not being varied (i.e. not on the x or y-axis) is fixed at 1 (a,e), 3 (b,f), or 5 (c,g). The first row (a,b,c) varies the parasites effect on development time (x-axis) and fecundity (y-axis) with the parasites effect on mortality changing from 1, 3 to 5 from left to right. The third row (e,f,g) show effects of fecundity (x-axis) and effects of mortality (y-axis) with effects on development time being 1,3, and 5 from left to right. The figure in the second row is a three dimensional depiction of the contour lines with effects on mortality (z-axis), on development time (y-axis) and on fecundity (x-axis). Notice that the effect on development time (y-axis) has been rotated so that the it goes from largest to smallest. Lines distinguish between different outcome categories. For example, yellow is when the proportion of *Ae. albopictus* exceeds 0.8.

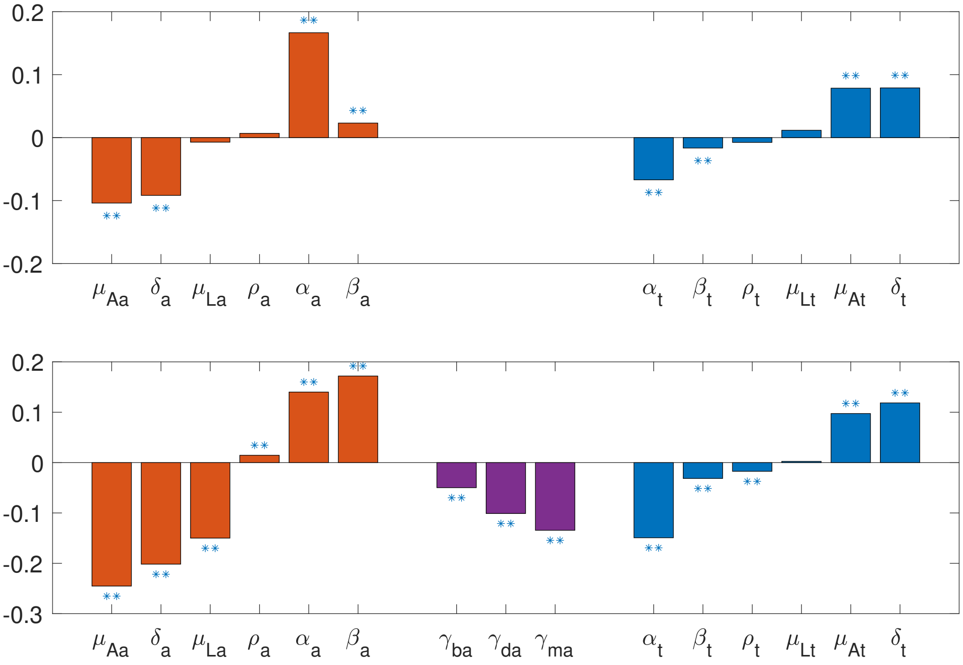

Figure 5: **Partial rank correlation coefficients of the parameter values with the final proportion of *Ae. albopictus* at 2000 days.** The stars indicate significance with a p-value less than 0.00001. The top plot shows the PRCC with the LHS with all parasitism parameters set to one ($\gamma_{d_a} = \gamma_{m_a}, \gamma_{b_a} = 1$), indicating no parasitism. The bottom plot is the PRCC with variation of parasitism included in the LHS for *Ae. albopictus* Parasitism on *Ae. triseriatus* is high. Red bars (left side) are parameters associated with *Ae. albopictus* and blue bars (right side) with *Ae. triseriatus*. Parasitism parameters on *Ae. albopictus* , which only occur in the bottom plot, are shown by purple bars (middle). For each grouping, parameters are ordered from least to greatest effect when including parasitism.
